# Effect of Wine Lees as Alternative Antioxidants on Physicochemical and Sensorial Composition of Deer Burgers Stored during Chilled Storage

**DOI:** 10.3390/antiox9080687

**Published:** 2020-08-02

**Authors:** Marina Alarcón, Manuel López-Viñas, María Soledad Pérez-Coello, María Consuelo Díaz-Maroto, María Elena Alañón, Almudena Soriano

**Affiliations:** 1 Area of Food Technology, Faculty of Chemical Sciences and Technologies, University of Castilla-La Mancha, Avda. Camilo José Cela 10, 13071 Ciudad Real, Spain; marina.ahernandez@uclm.es (M.A.); manuel.lvinas@uclm.es (M.L.-V.); soledad.perez@uclm.es (M.S.P.-C.); 2 Regional Institute for Applied Scientific Research (IRICA), University of Castilla-La Mancha, Avda. Camilo José Cela 10, 13071 Ciudad Real, Spain; mariaconsuelo.diaz@uclm.es; 3 Area of Food Technology, Higher Technical School of Agronomic Engineering, University of Castilla-La Mancha, Ronda de Calatrava 7, 13071 Ciudad Real, Spain; mariaelena.alanon@uclm.es

**Keywords:** wine lees, deer burger, protein and lipid oxidation, antioxidant, antimicrobial, volatile compounds, sensorial characteristics

## Abstract

Wine lees from two grape varieties (*Vitis vinifera* L. Cv. “verdejo” and “palomino”) were studied as natural preservatives in deer burgers compared with the traditional additive sodium ascorbate. Burgers packed in modified atmosphere packaging and stored in refrigeration were analyzed at 0, 4, 8, and 12 days. The addition of lees (2.5% and 5%) produced a reduction of pH and variations in color (L* and a*), higher antioxidant capacity and phenolic content, lower lipid and protein oxidation, and the inhibition of psychotrophic aerobic bacteria and enterobacteria during the storage time. Likewise, burgers with lees kept the aldehydes concentration (volatile compounds indicators of lipid oxidation) over storage time, while esters, acids, and other compounds, previously present in lees, increased. These changes provided new odor and taste attributes like wine, bakery, and raisin notes. Therefore, the addition of wine lees had an antioxidant and antimicrobial effect and produced new sensory attributes in deer burgers.

## 1. Introduction

Meat and meat products are widely considered as a main part of the human diet since they are a resource of bioavailable amino acids, proteins, lipids, minerals, and vitamins. Consumers demand meat products because they are easy to store and cook. In general, they are elaborated mainly with livestock animals like pork, beef, or poultry, but today, consumers look for other unusual species. In this sense, venison meat from wild red deer is a viable alternative to traditional meats in an increasingly demanding market. It is considered a “natural” meat, obtained from free-range animals and free of hormone, antibiotics, and other products. Also, its nutritional quality allows consumers to follow a healthy diet due to its low fat and saturated fatty acid content and high protein and trace mineral content [1]. Despite not being widely produced and consumed worldwide, the demand of this meat has increased in Europe over the last few decades; even consumers pay a higher price for it [2]. There is a wide variety of products elaborated with meat; nevertheless, burgers are one of the most popular due to the combination of convenience and low price, along with sensorial properties. However, the grinding process and their high fat content make burgers more susceptible to oxidation and microbial spoilage due to the breakdown of muscle structure. In addition, different processing stages of burgers and the modified atmosphere packaging (MAP) rich in oxygen facilitate the oxidative process [3]. Therefore, the use of additives to prevent deterioration of meat products is necessary to extend their shelf life.

Although the food industry has commonly employed synthetic additives to maintain the quality of meat products, consumers prefer the use of natural preservatives. Essential oils, aqueous extracts, powders, and other plant products have been proposed as natural additives due to their phenolic content being responsible for the antioxidant and antimicrobial capacities [4]. However, the occurrence of these valuable compounds can also be found in the large amounts of waste generated by food industries. Therefore, the revalorization of agro-food byproducts drives economic advantages and provides new commercial utilities because of their content in bioactive compounds [5].

In particular, the wine industry produces 2–3 million tons of waste products per year only in Spain, which need to be processed [6]. Consequently, some environmentally friendly technologies have appeared to revalorize these winemaking residues with a high content of bioactive compounds [7]. Wine lees are a water-waste sediment composed of solid and liquid fractions, deposited in the bottom of the tanks or barrels during the vinification process. They mostly contain microbial biomass, metabolites, and phenolic compounds, which leads a source of wide interesting products [8]. Due to their composition, wine lees can be used in food, pharmaceutical, and cosmetic industries because of their recognized antioxidant, antimicrobial, cardio protective, or anti-inflammatory properties [9].

Inactive dry yeasts (IDY) from *Saccharomyces cerevisiae* have been proposed in the food industry as natural antioxidants [10], but few studies have employed natural wine lees in useful applications as functional additive in foodstuff [11,12]. Nevertheless, other winery byproducts such as seed and grape pomace extracts have been used as alternative antioxidants in different meat products [13,14,15].

Taking into account the functional properties of wine lees, this research has been carried out to find an alternative to traditional and synthetic additives in the meat industry. With this aim, the antioxidant and antimicrobial efficiency of wine lees added to deer burgers packed in MAP to enhance their self-life was checked. Also, changes in the chemical and sensorial properties of burgers were studied.

## 2. Materials and Methods

### 2.1. Raw Materials and Ingredients

Burgers were prepared employing lean from wild red deer (*Cervus elaphus*) and pork fat. Boneless legs of wild stags hunted in “montería”, following Spanish Law 2/1993, were used. Fat from female pigs of the same crossbreed were used. These raw materials were obtained from a local supplier (Ciudad Real, Spain). Other ingredients used in the formulation were mineral water, sodium chloride, and sodium ascorbate (Panreac Química, S.A., Barcelona, Spain). Lyophilized lees from the fermentation by *Saccharomyces cerevisiae* of must from “verdejo” (Lv) and “palomino” (Lp) grape varieties were employed. Both types of wine lees were supplied by the Department of Analytical Chemistry of University of Cádiz (Spain).

### 2.2. Preparation of Burgers and Storage Conditions

Different types of burgers were prepared. Control burgers (C) were obtained by mixing the following amounts (*w/w*) of ingredients: 70% deer lean, 15% pork fat, 14% mineral water, 1% sodium chloride, and 400 ppm of sodium ascorbate. Deer lean and pork fat were previously minced separately in an Unger W-98 table-top mincer (Andher, Alcázar de San Juan, Ciudad Real, Spain) through an 8 mm plate. Sodium chloride and sodium ascorbate were dissolved in water, added to the ground raw materials and homogenized manually for 3 min. Burgers with wine lees were made using the base formulation of C but adding “verdejo” and “palomino” lees in two proportions (*w/w*), 2.5% (Lva, Lpa) and 5% (Lvb, Lvp), instead of sodium ascorbate. Deer burgers (100 g) were formed employing a conventional burger-maker. Burgers were individually packed in thermoformed polypropylene plastic trays (16.45 × 11.85 cm, 460 mL capacity) and heat sealed by a transparent top film (polyamide 15 and polypropylene 50). High-oxygen atmosphere composed of 80% O_2_ and 20% CO_2_ (Aligal 27, Air Liquide, Madrid, Spain) was applied using an Orved packing machine (Andher). In order to simulate the supermarket conditions, samples were kept in a refrigerated display with transparent glass doors and white light lamps of 150 lumens (Pecomark, Barcelona, Spain) for 12 days. Storage was carried out at 4 °C and 12 h/day of light exposure. Three burgers of each treatment were taken at 0, 4, 8, and 12 days of chilled storage for the microbial and physicochemical analyses. Volatile compound analysis was performed at days 0 and 12. Sensorial analysis was made only before storage.

### 2.3. Physicochemical Parameters

The pH of burgers was assessed directly in the sample in two different points using a Crison 2002 pH-meter (Crison Instruments S.A., Barcelona, Spain) equipped with a glass electrode probe (model Crison 52-32; Crison Instruments S.A.). Moisture was measured in each sample by oven-drying in duplicate (ISO, 1973). Color parameters L* (lightness), a* (redness), and b* (yellowness) were evaluated in three different points after 30 min blooming time using a Minolta CM-2600d portable colorimeter (Konica Minolta, Osaka, Japan). Illuminant D65 and 10° standard observer were fitted. To determine the total phenolic content (TPC) and radical scavenging activity, a previously sample extraction in duplicate was carried out, and then, for the consequent analysis by Folin index, DPPH and ABTS methods was applied [16]. Lipid oxidation was evaluated in duplicate in each sample by TBARs analysis according to Serrano, Cofrades, and Jiménez-Colmenero (2006) [17]. Protein oxidation was determined by quantifying the protein carbonyl amount using the method described by Ganhão, Morcuende, and Estévez (2010) [18] in duplicate in each sample.

### 2.4. Microbiological Quantification

Microbiological quantification was carried out by bacterial counts on selective media and conditions. Total viable counts (TVC) and psychotrophic aerobic counts were performed using plate count agar (PCA; ThermoFisher Scientific, Waltham, MA, USA), being incubated at 30 °C for three days and at 4 °C for six days, respectively. *Enterobacteriaceae* were enumerated on Violet Red Bile Glucose Agar (VRBGA; ThermoFisher Scientific) by anaerobic incubation, getting it by anaerobic gas generating sachets (AnaeroGen TM, ThermoFisher Scientific), at 37 °C for 1 day. Lactic acid bacteria (LAB) were analyzed on de-Man Rugosa Sharpe agar (MRS; Pronadisa, Madrid, Spain) at pH 5.7, incubated under anaerobic conditions (AnaeroGen TM, ThermoFisher Scientific) at 30 °C for two days. Yeasts were incubated in Rose Bangal agar (Pronadisa) containing chloramphenicol (0.1 g/L) at 30 °C for two days. Microbial analysis was carried out in duplicate in each sample.

### 2.5. Extraction and Analysis of Volatile Compounds

Volatile compounds of samples were analyzed by headspace/solid phase microextraction (HS-SPME) and gas chromatography coupled to mass spectrometry (GC-MS) following the method described by Soriano et al., 2018 [16]. Extraction was conducted using a fiber of DVB/CAR/PDMS 50/30 μm (Supelco Co., Bellefonte, PA, USA). GC-MS analysis was carried out on a 6890 N Agilent gas chromatograph coupled to a 5973 N Agilent Mass Detector, equipped with a polar DB-WAX ultra-inert column, 60 m × 0.25 mm i.d.; 0.25 μm film thickness (Agilent Technologies, Inc.). The carrier gas was helium at 1 mL/min and the oven temperature was programmed to start at 35 °C/3 min, increasing 2 °C/min to 180 °C, and then 10 °C/min to 220 °C (15 min). The MS worked in the electron impact mode with electron energy of 70 eV, the ion source temperature was 230 °C and the scanning was made from 45 a.m.u. to 550 a.m.u.

Identification of volatile components was performed compared to the authentic standards from Sigma-Aldrich (Tres Cantos, Madrid, Spain). The tentative identification of compounds for which it was not possible to find reference volatiles was carried out by comparison of their mass spectra with spectral data from Wiley G 1035 A and NBS75K libraries and by the linear retention index (LRI) comparison. Semiquantitative analysis of the compounds was performed assuming that component response factors were the same as the response factor for the internal standard. The concentration of each volatile compound was expressed in ng/g_DM_ [19].

### 2.6. Sensorial Analysis

A quantitative descriptive analysis (QDA) was carried out in samples according to UNE-EN ISO 13299:2016. Burgers at day 0 were analyzed before and after cooking. Analysis was performed by a 10-member trained panel, composed of seven women and three men aged between 22 and 60 years of age and staff of the Food Technology Area of the University of Castilla-La Mancha. The panel had previous experience in meat product analysis, including burgers. One training session was held, employing commercial deer burgers from a local supplier (Ciudad Real, Spain). The analysis was executed in a tasting room designed and equipped in accordance with UNE-EN ISO 8589:2010. Prior to QDA, a session was developed with lees at work concentrations (2.5% and 5% in water) in order to generate sensory attributes. The panel evaluated appearance and odor attributes in raw burgers and appearance, odor, taste, and texture attributes in cooked burgers. Attributes were assessed using 10-cm non-structured line scales with two anchors at the ends. Burgers were cooked on a griddle until reaching an internal temperature of pasteurization (72–73 °C). Raw samples were presented in dishes at 20–22 °C, and cooked samples in individual pieces covered with aluminum foil at 50 °C. All samples were coded labelled (number-letter-number). Sensorial analysis was carried out in a single session.

### 2.7. Chemical Characterization of Wine Lees

Table 1 shows the chemical characterization of wine lees, in terms of volatile and phenolic composition. Volatile compounds were evaluated employing the same methodology and equipment as in meat samples but taking 2.5 g of lees. In order to determine the phenolic profile, a previous lees extraction was carried out according to the method described by Soriano et al., 2018 [16] used to evaluate the TPC and antioxidant activity. Undiluted lees extracts were employed to test hydroxycinnamic acid derivates (HCA) and flavonols following the method proposed by Mazza, Fukumoto, Delaquis, Girard, and Ewert (1999) [20] and catechins by means the method described by Vivas, Glories, Laguna, Saucier, and Augustin (1994) [21].

### 2.8. Statistical Analysis

In order to determine the influence of wine lees addition or storage time on the different variables a one-way analysis of variance (ANOVA) and a subsequent post hoc Student–Newman–Keuls test were carried out. In the case of volatile and phenolic composition of lees, a t Student test was executed to observe the significant differences between type of lees and in volatile compounds between day 0 and 12 in burgers. A significant difference was considered when *p* < 0.05. Principal component analysis (PCA) was applied to find the relationships among different type of sample and times of storage regarding volatile composition. Statistical analysis was carried out using the IBM SPSS 24.0 software for windows statistical package (SPSS, Inc., Chicago, IL, USA).

## 3. Results and Discussion

### 3.1. Physicochemical Parameters and Color

Table 2 shows the results of pH, moisture, and color of burgers during chilled storage. A significant reduction of pH was appreciated in all samples during storage, possibly because of the generation of lactic acid by LAB, and the carbonic acid formation due to the reaction of the CO_2_ of MAP and the water of the samples. Likewise, lees addition in burgers caused a decrease of the pH, probably due to the acidity of lees extracts.

The moisture content was not significantly affected during storage. On the other hand, burgers with lees showed lower moisture values than control, mainly those with the highest concentration of lees (Lvb and Lpb).

Regarding color parameters, lees addition caused few variations in the component L*, although at the end of storage, control burgers presented the highest values of L*, indicating the surface discoloration of meat during chilled storage [16]. Component a* decreased significantly over time due to loss of red color of fresh meat during storage, probably due to the myoglobin (red-purple color) oxidation to metmyoglobin (brown red color) [22]. Burgers with lees preserved their color better than control samples, and the decrease in component a* was less. This fact has been previously observed with different natural antioxidants [16,23,24]. The parameter b* presented an unclear evolution respect to storage time, as shown in other studies about deer meat packaged in MAP [25].

### 3.2. Microbiological Quantification

**Figure 1:**
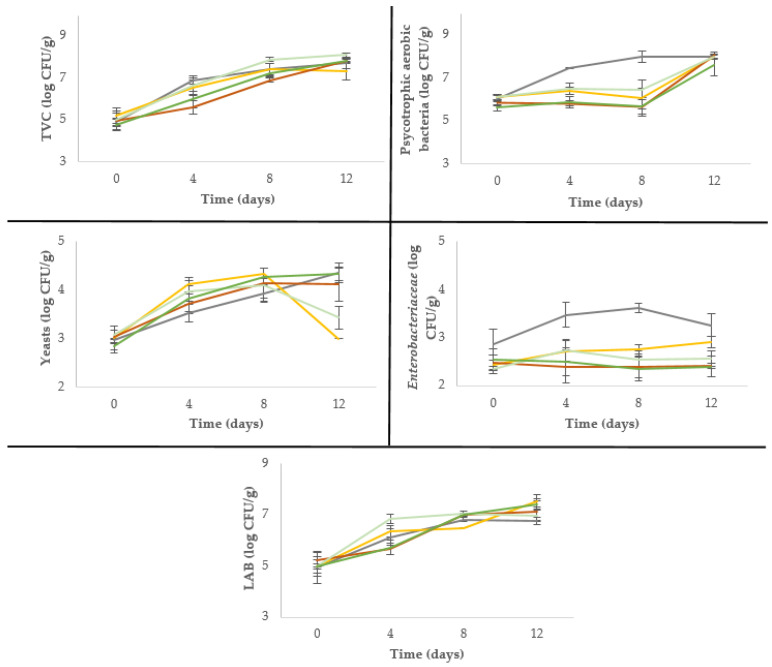
Modifications in microbial counts of deer burgers packed in MAP during chilled storage. Symbols: 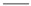 C; 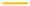 Lva; 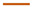 Lvb; 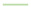 Lpa; 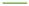 Lpb. C: sodium ascorbate control; Lva: 2.5% “verdejo” lees; Lvb: 5% “verdejo” lees; Lpa: 2.5% “palomino” lees; Lpb: 5% “palomino” lees.

The microbiological counts of burgers packed in MAP during chilled storage are shown in Figure 1. Initial counts were higher than in burgers made with other animal species due to the type of evisceration and manage of carcass, which is performed in the field instead in a slaughterhouse [26]. In this sense, initial counts around 4.0–5.1 log CFU/g of TVC, 3.0–4.5 log CFU/g of psychotrophic, and 2.0–4.5 log CFU/g of LAB were found in pork burgers [23].

Freshly made burgers showed a TVC of 4.8–5.2 log CFU/g, but a significant increase (*p* < 0.001) was observed during storage in all samples. In general, wine lees addition did not affect TVC (*p* > 0.05). In this sense, other natural extracts (chestnut and seaweed) did not influenced TVC throughout storage, while tea and grape extracts and tomato powder led to a decrease in pork burgers [23,27]. Furthermore, it was observed that chilled storage caused a progressive increase (*p* < 0.001) in psychotrophic aerobic bacteria counts, especially in control burgers, reaching values of 8 log CFU/g, while burgers with lees kept the initial counts during the first eight days of storage (5.7–6.5 log CFU/g). *Enterobacteriaceae* counts exhibited a significant increase (*p* < 0.001) over time in control burgers reaching 3.3 log CFU/g at the end of storage. Burgers with 2.5% wine lees (Lva and Lpa) showed this growth pattern but lower counts (2.6–2.9 log CFU/g). No growth was observed in burgers with 5% lees (Lvb and Lpb) maintaining 2.4–2.6 log CFU/g during storage.

The inhibition of psychotrophic and *Enterobacteriaceae* growth was also observed in other studies where the ability of wine pomace to extend the shelf life was tested [13,15].

It is known that LAB constitute a wide part of the natural microbiota of meat packed in MAP [28]. A significant increase (*p* < 0.001) in LAB counts was observed during chilled storage in all samples, however no significant effect of wine lees addition was observed after 12 days of storage.

Similarly, yeast counts increased with storage time, although, in the case of burgers with 2.5% lees (Lva and Lpa), the final yeast counts were significantly lower (*p* < 0.001) than control.

### 3.3. Total Phenolic Content (TPC) and Radical Scavenging Activity (ABTS, DPPH)

**Figure 2:**
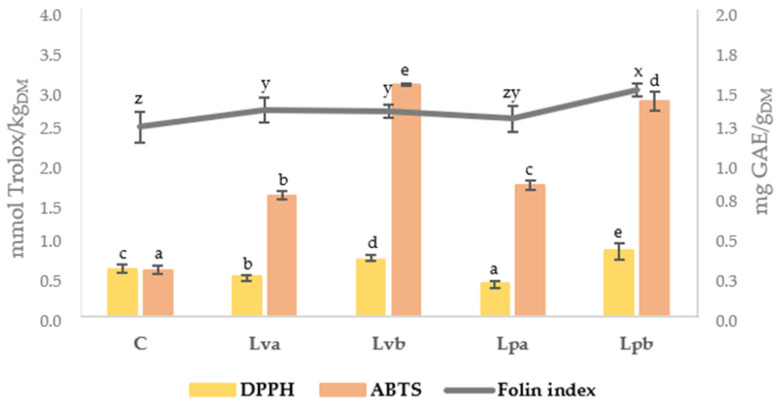
Total phenol content (Folin index) and radical scavenging activity determined by DPPH and ABTS methods of deer burgers after 12 days of chilled storage. C: sodium ascorbate control; Lva: 2.5% “verdejo” lees; Lvb: 5% “verdejo” lees; Lpa: 2.5% “palomino” lees; Lpb: 5% “palomino” lees; DM: dry matter. Different lowercase letters appearing in the same method for determining antioxidant activity (a–d) and Folin index (z–x) indicate significant differences (*p* < 0.05) between samples.

Burgers with wine lees showed significantly higher values of TPC (mg GAE/g_DM_) than burgers with sodium ascorbate (Figure 2) due to the phenolic composition of the lees (Table 1). Despite the highest phenolic content detected in lees from Palomino variety, only significant differences were found in burgers skyped with the greatest lees concentration. An increase in TPC was also reported in pork burgers with oak wood extracts added as a natural antioxidant from 0.49 mg GAE/g_DM_ in control with sodium ascorbate to 1.75 mg GAE/g_DM_ in burgers with 1% oak wood extract at 12 days of storage [16].

After 12 days of storage, burgers with 5% wine lees (Lvb and Lpb) showed the highest values of radical scavenging activity, tested by both methods, but ABTS was more efficient to highlight the differences. Other authors have also observed a greater antioxidant activity using natural preservatives instead of ascorbic acid [29].

### 3.4. Lipid and Protein Oxidation

Lipid oxidation was measured by thiobarbituric acid-reactive substances (TBARs) analysis, which determines the content of secondary lipid oxidation products responsible of the meat off-flavors. TBARs values increased over the storage time in all burger types (Table 3).

This rise was also observed in other studies with pork and beef burgers [13,30], and it reveals the instability of minced products even though they are stored in MAP conditions. Control samples presented the greater increase, reaching the highest values at days 8 and 12. Therefore, the wine lees prevented lipid oxidation, mainly after four days of storage, being more effective than sodium ascorbate. This property could be due to their high phenolic content and scavenging activity (Figure 2), which was also observed by other authors in ice creams [12]. TBARs values in control samples after eight days of storage (7.39 mg MDA/kg) exceeded 5 mg MDA/kg, concentration established as acceptability limit from a sensorial point of view [31]. However, samples with lees did not reach this limit concentration during storage, showing a maximum value of 4.06 mg MDA/kg (Lpa).

Carbonyls are one of the main products of oxidized proteins and they have been widely used as indicators of protein oxidation in food and biological systems [32]. Table 3 shows the results of protein oxidation in deer burgers. All samples showed a significant increase of hydrazones over time, mainly between days 4 and 8. The use of wine lees significantly decreased the carbonyl content only at the beginning of storage. Other authors also reported a significant reduction of protein oxidation employing oenological by-products such as wine pomace in different meat products [23,33]. This protective effect was in agreement with TBARs values, showing the link between lipid and protein oxidation [34].

No effect was observed due to the concentration or type of lees used, despite the higher content of phenolic compounds in “palomino” lees (Table 1).

### 3.5. Volatile Compounds

Lipid oxidation produces different volatile organic compounds (VOCs), which give rancid and unpleasant flavors and decrease the sensorial quality of meat products [35]. Moreover, the use of high oxygen atmosphere to prolong the shelf life of raw meat promotes oxidative changes and provides excellent conditions for the development of VOCs in meat during storage [36].

To the best of our knowledge, the volatile profile of wild red deer meat has been little studied, only being reported in smoked dried meat [19] and raw farm meat [37]. In our study, a total of 95 volatile compounds were identified and quantified in raw deer burgers, including aldehydes, ketones, alcohols, hydrocarbons, acids, esters, benzenic compounds, sulfur compounds, and furans (Supplementary data, Table S1). Table 4 shows the most representative VOCs in the deer burger flavor with and without the addition of wine lees, together with the total amount of volatiles grouped into chemical families, at 0 and 12 days of storage.

Aldehydes are indicators of raw and cooked meat rancid odors due to their high concentration and low odor threshold [38] and show a good correlation with TBARs values [39]. Some aliphatic saturated aldehydes like hexanal, octanal and nonanal formed from the oxidation of linoleic and oleic acids showed the highest concentrations in all the samples. These compounds have been identified as markers of lipid oxidation [40,41]. Also, unsaturated aldehydes (2-heptenal, 2-octenal, 2-nonenal, 2,4-nonadienal, 2,4-decadienal), formed from polyunsaturated fatty acids (PUFA), were found in high quantities. They could have an important role in the flavor of burgers due to their low odor thresholds. A high level of PUFA was found in the intramuscular fat of deer meats, and autoxidation could be initiated more readily in this type of meat [42]. Some authors observed aldehydes inhibition in different meat products using natural antioxidants [43,44]. In our case, the total quantity of aldehydes increased in control samples during chilled storage in MAP; however, in the burgers with lees, it remained constant (except in the case of the Lpb sample). Certain aldehydes such as hexanal, octanal, nonanal, and (E)-2-nonenal presented less concentration in Lva burgers at the end of storage.

Other compounds also originated by lipid oxidation such as hydrocarbons and ketones showed less changes because of the storage and lees addition [45]. Control samples maintained the ketones concentration over time, while the addition of lees induced a small increase in most of these compounds at day 12. No differences were observed between samples at day 0. Despite their low concentration in samples, ketones may provide pungent and rancid off-odors to meat products [41]. 1-Octen-3-one and its related alcohol, 1-octen-3-ol, with mushroom aroma, were also present in all burgers (control samples and those treated with lees). 1-Octen-3-ol, with an important role in the flavor of meat products [46], showed a significant increase during storage.

Hydrocarbons are considered the main volatile compounds formed via lipid oxidation in deer cecina (Spanish smoked dry-cured meat product) [36], but they have no significant impact on flavor.

Aliphatic alcohols suffered a considerable increase at the end of storage, especially in the case of 1-hexanol, which was the predominant alcohol in deer burgers after 12 days of chilled storage, followed by 1-octen-3-ol. It is remarkable that at the end of storage, many alcohols like 1-pentanol, 4-heptanol, 2-ethyl-1-hexanol, and 1-octanol showed significantly lower concentrations in burgers with wine lees than in control samples.

Aliphatic acids detected in deer burgers were produced mainly by the degradation of triglycerides and phospholipids, while esters could be generated from the esterification of several alcohols and carboxylic acids in meat products during storage [46,47].

Esters provide fruity notes, mainly those derived from short-chain acids, and slight fatty odor from long-chain acids. The addition of lees caused a high increase in the total acids and esters of burgers since these compounds are formed during wine fermentation and they are present in wine lees (Table 1). Among them, hexanoic, octanoic, and decanoic acids and their esters were the most abundant in all the samples. However, although an increase of total acids was observed in control burger after 12 days of storage, ester concentrations were significantly reduced in burgers with less, especially in the case of the ethyl decanoate.

Storage led to an increase in the total of benzene compounds in the control and lees samples. Some of them, such as benzaldehyde and phenylethyl alcohol, derived from wine fermentation, increased their concentrations due to the use of lees. These compounds could improve the aroma of deer burgers with characteristic notes, while others, such as p-cymene or trimethyl benzene, with high odor thresholds, may not significantly affect the aroma of meat products [47].

Sulfur compounds can arise from the catabolism of sulfur-containing amino acids or generated by microorganisms [46,48]. They are sensorial active compounds of meat products with pleasant notes when in moderate concentrations [41]. Only two sulfur compounds were detected in samples—carbon disulfide and 5-methyl thiazole, the last one detected after 12 days of storage at greater concentrations in samples Lvb and Lpb.

Furthermore, some furanic compounds were identified, 2-pentyl furan being the most abundant, although small variations were observed with respect to control samples during storage.

**Figure 3:**
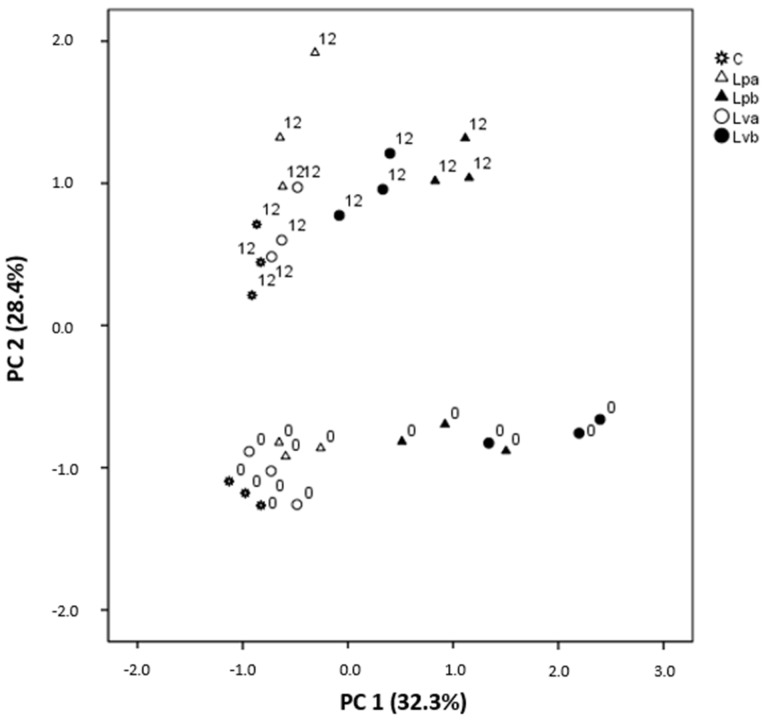
Distribution of deer burgers during chilled storage in the space defined by the first two Principal Components (PC1 and PC2). The selected variable was the volatile composition analyzed by SPME-GC-MS. C: sodium ascorbate control; Lva: 2.5% “verdejo” lees; Lvb: 5% “verdejo” lees; Lpa: 2.5% “palomino” lees; Lpb: 5% “palomino” lees.

In order to highlight the differences in deer burgers based on their volatile composition, PCA was applied to the completely volatile data set (Table S1). Table 5 shows the most correlated volatile compounds with the two first principal components and their loadings. Sample distribution in the plane formed by the first two main components is shown in Figure 3. Principal Component 1 (PC 1) was positively related with several esters such as ethyl decanoate, 3-methylbutyl decanoate, and 3-methylbutyl octanoate and acids like octanoic acid and phenylethyl alcohol (Table 5). Samples with the highest amount of these compounds were burgers with wine lees (Lvb and Lpb) at days 0 and 12. On the other hand, Principal Component 2 (PC 2) clearly separated the samples based on storage time, and it was positively correlated with benzaldehyde; several alcohols such as 1-hexanol, (E)-2-octen-1-ol and 1-octen-3-ol; esters like ethyl hexanoate; and acids including pentanoic and hexanoic acids. Therefore, ester concentration was mainly related with the type of sample, increasing with the amount of lees, while the storage time generally produced an increase in the concentration of alcohols and acids.

### 3.6. Sensorial Analysis

**Figure 4:**
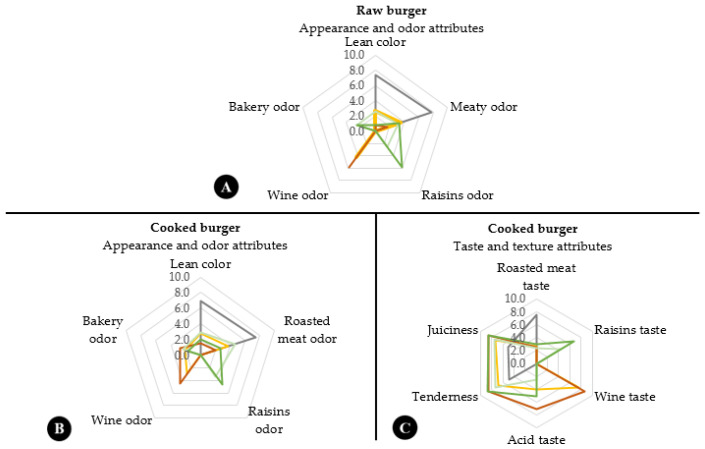
Quantitative descriptive analysis of raw and cooked venison burgers. Symbols: 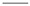 C; 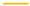 Lva; 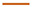 Lvb; 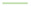 Lpa; 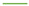 Lpb. C: sodium ascorbate control; Lva: 2.5% “verdejo” lees; Lvb: 5% “verdejo” lees; Lpa: 2.5% “palomino” lees; Lpb: 5% “palomino” lees. (**A**) Raw burgers: Appearance and odor attributes; (**B**) Cooked burgers: Appearance and odor attributes; (**C**) Cooked burgers: Taste and texture attributes.

Extracts addition could have a negative effect on the appearance, aroma, and taste, especially when their concentrations are high [49]. To determine the influence of lees addition on sensorial characteristics, QDA was carried out in raw and cooked burgers at the beginning of storage (day 0). In raw samples, appearance and odor attributes were evaluated (Figure 4A). The characteristic purple red color decreased in samples with lees. The higher lees concentration, the lower color scores (0 = brown, 10 = bright red). As it was observed in instrumental color, a* values were lower in samples with lees (Table 2). Regarding odor attributes, samples with wine lees showed new particular odors that attenuated the typical meaty odor. These new attributes were assessed as “wine” in samples with “verdejo” lees (Lva, Lvb) and “bakery” and “raisins” for samples with “palomino” lees (Lpa, Lpb). The color alteration and typical sensorial changes were also observed by other authors after adding grape seed and peel extracts in a chicken meat product [14].

Regarding cooked burgers (Figure 4B,C), control samples were characterized by a higher rose color and “roasted meat” odor. However, in burgers with lees, the new taste attributes also appeared—“raisins” (Lpa and Lpb) and “wine” (Lva and Lvb), being their intensities proportional to the lees concentration. These new attributes were considered as pleasant at low intensities and could be associated with the higher amounts of esters, acids, and benzenic compounds detected in the volatile profile of burgers with lees. They were positively appreciated by the panelists. The use of wood extract as natural antioxidants modified slightly and pleasantly the typical aroma of raw pork patties [16]. Acid taste appeared only in samples with lees. Moreover, lees addition offered more juiciness and tenderness to deer burgers that increased with the lees concentration.

## 4. Conclusions

The replacement of sodium ascorbate by lees (2.5% and 5%) causes a reduction of the pH and modifies color parameters (increase of L* and reduction of a*), protecting the discoloration of deer burgers during a storage time. Moreover, lees reduce the psychotrophic aerobic bacteria counts during the first eight days of storage in MAP, as well as the *Enterobacteriaceae* counts, from the moment of their addition. On the other hand, lees provide a higher antioxidant capacity and phenolic content than sodium ascorbate, mainly at the highest concentrations. This fact results in a higher protection against lipid and protein oxidation of burgers during the storage in MAP. Although most of the volatile compounds increased at the end of storage in all samples, aldehydes that are markers of lipid oxidation, maintained their concentration in burgers with lees. However, some compounds present in lees such as esters, acids, and benzenic compounds greatly contribute to increase the volatile fraction of the experimental burgers, providing them new odor and taste attributes.

Therefore, wine lees from the oenological industry could be a good alternative as natural preservatives in meat products. A widespread use would contribute to the revalorization of this byproduct.

## Figures and Tables

**Table 1:** Volatile and phenolic composition of wine lees (n = 2) used in the elaboration of the burgers (mean ± standard deviation).

	Lv	Lp
Volatile Compounds (µg/g_DM_)		
∑ esters	199.35 ± 32.70	199.60 ± 62.36
∑ acids	26.77 ± 8.46	18.56 ± 7.14
∑ alcohols	1.34 ± 0.43	1.27 ± 0.55
∑ aldehydes	0.65 ± 0.08	0.56 ± 0.19
∑ terpenes and C_13_ norisoprenoids	0.40 ± 0.05 ^a^	0.67 ± 0.07 ^b^
∑ furanic compounds	0.64 ± 0.25	0.47 ± 0.19
Phenolic Composition		
Hydroxycinnamic acid derivates (mg HCA/100 g_DM_)	1.53 ± 0.11 ^a^	2.59 ± 0.12 ^b^
Flavonols (mg quercetin/100 g_DM_)	1.45 ± 0.12 ^a^	2.54 ± 0.12 ^b^
Catechins (mg catechin/100 g_DM_)	15.09 ± 1.36 ^a^	30.24 ± 1.71 ^b^

Lv: “verdejo” lees; Lp: “palomino” lees; DM: dry matter. Means within a row with different superscript letters (^a,b^) are significantly different (*p* < 0.05) as a function of type of lees.

**Table 2:** Physicochemical and color parameters (mean ± standard deviation) of deer burgers during chilled storage.

	C	Lva	Lvb	Lpa	Lpb
pH					
Day 0	5.66 ± 0.01 ^d,w^	5.22 ± 0.08 ^c,x^	4.78 ± 0.02 ^a,y^	5.16 ± 0.02 ^c,w^	4.91 ± 0.02 ^b,x^
4	5.45 ± 0.01 ^c,x^	5.14 ± 0.02 ^b,x^	4.94 ± 0.07 ^a,x^	5.12 ± 0.01 ^b,x^	4.90 ± 0.07 ^a,x^
8	5.19 ± 0.02 ^c,y^	4.86 ± 0.02 ^b,y^	4.70 ± 0.07 ^a,y^	4.80 ± 0.02 ^a,b,y^	4.73 ± 0.08 ^a,y^
12	5.03 ± 0.02 ^c,z^	4.73 ± 0.04 ^b,z^	4.54 ± 0.02 ^a,z^	4.75 ± 0.01 ^b,z^	4.55 ± 0.05 ^a,z^
Moisture (%)					
Day 0	69.65 ± 1.02 ^c^	66.37 ± 0.00 ^a,b^	65.21 ± 0.83 ^a^	67.71 ± 0.90 ^b^	65.04 ± 0.56 ^a^
4	67.22 ± 1.03	67.02 ± 0.68	66.36 ± 0.33	68.10 ± 0.68	66.28 ± 0.83
8	67.85 ± 0.65 ^b^	66.84 ± 0.29 ^a,b^	65.14 ± 1.41 ^a^	66.20 ± 0.88 ^a,b^	65.70 ± 0.61 ^a^
12	69.63 ± 1.47 ^c^	67.18 ± 0.26 ^b^	64.86 ± 0.65 ^a^	67.00 ± 0.57 ^b^	66.13 ± 0.12 ^a,b^
L*					
Day 0	47.60 ± 0.75 ^a,z^	48.72 ± 0.34 ^a,z,y^	51.28 ± 1.44 ^b,z,y^	47.21 ± 1.73 ^a,z^	51.44 ± 0.88 ^b,y^
4	49.49 ± 1.36 ^a,b,z^	50.79 ± 1.04 ^b,y^	52.94 ± 0.46 ^c,y^	48.73 ± 0.26 ^a,z,y^	51.13 ± 0.72 ^b,y^
8	48.60 ± 2.08 ^z^	47.44 ± 1.27 ^z^	48.80 ± 1.04 ^z^	47.14 ± 1.56 ^z^	48.48 ± 1.50 ^z^
12	53.63 ± 2.58 ^y^	50.32 ± 0.66 ^y^	52.67 ± 2.34 ^y^	50.64 ± 0.83 ^y^	52.57 ± 0.77 ^y^
a*					
Day 0	13.30 ± 0.96 ^c,x^	7.27 ± 0.41 ^a,b,y^	6.38 ± 0.18 ^a,y^	7.60 ± 0.22 ^b,y^	6.26 ± 0.41 ^a,y^
4	7.90 ± 0.60 ^c,y^	5.41 ± 0.51 ^a,b,z^	5.05 ± 0.31 ^a,z^	6.24 ± 0.25 ^b,z^	6.00 ± 0.12 ^b,y^
8	4.90 ± 0.53 ^z^	4.85 ± 1.00 ^z^	4.95 ± 0.09 ^z^	6.15 ± 0.45 ^z^	4.40 ± 1.10 ^z^
12	4.65 ± 0.15 ^a,z^	6.00 ± 0.25 ^b,z^	4.99 ± 0.91 ^a,b,z^	5.62 ± 0.46 ^a,b,z^	5.46 ± 0.07 ^a,b,z,y^
b*					
Day 0	19.75 ± 0.58 ^y^	19.77 ± 0.56 ^y^	20.13 ± 0.86 ^z^	19.66 ± 0.93 ^z^	20.82 ± 0.73 ^y^
4	17.64 ± 0.64 ^a,b,z^	17.07 ± 0.77 ^a,z^	19.14 ± 0.52 ^b,z^	18.18 ± 0.59 ^a,b,z^	18.96 ± 0.47 ^b,z^
8	27.18 ± 0.53 ^b,x^	26.27 ± 0.47 ^b,x^	27.13 ± 0.63 ^b,y^	24.69 ± 0.61 ^a,y^	26.89 ± 0.80 ^b,x^
12	20.18 ± 0.61 ^b,y^	18.05 ± 0.47 ^a,z^	19.92 ± 0.77 ^b,z^	18.87 ± 0.59 ^a,b,z^	19.38 ± 0.40 ^b,z^

C: sodium ascorbate control; Lva: 2.5% “verdejo” lees; Lvb: 5% “verdejo” lees; Lpa: 2.5% “palomino” lees; Lpb: 5% “palomino” lees. Means within a row with different superscript letters (^a–d^) are significantly different (*p* < 0.05) as a function of sample. Means within a column with different superscript letters (^z–w^) are significantly different (*p* < 0.05) as a function of storage.

**Table 3:** Thiobarbituric acid-reactive substances (TBARs) and protein hydrazones (mean ± standard deviation) in deer burgers during chilled storage.

	C	Lva	Lvb	Lpa	Lpb
TBARs (mg MDA/kg)					
Day 0	0.93 ± 0.18 ^b,z^	0.54 ± 0.06 ^a,z^	0.58 ± 0.07 ^a,z^	1.25 ± 0.17 ^c,z^	0.92 ± 0.14 ^b,z^
4	2.95 ± 0.36 ^c,y^	2.32 ± 0.36 ^b,y^	1.77 ± 0.11 ^a,y^	3.05 ± 0.25 ^c,y^	1.90 ± 0.13 ^a,y^
8	6.42 ± 0.66 ^b,x^	3.15 ± 0.64 ^a,x^	2.93 ± 0.17 ^a,x^	3.20 ± 0.21 ^a,y^	2.50 ± 0.31 ^a,x^
12	7.39 ± 0.91 ^c,w^	3.67 ± 0.26 ^a,b,w^	3.01 ± 0.41 ^a,x^	4.06 ± 0.55 ^b,x^	2.91 ± 0.41 ^a,w^
Protein Oxidation (nmol hydrazone/mg protein)					
Day 0	4.21 ± 0.88 ^b,z^	2.24 ± 0.71 ^a,z^	2.63 ± 0.29 ^a,z^	2.32 ± 0.51 ^a,z^	1.98 ± 0.73 ^a,z^
4	4.82 ± 0.88 ^c,z^	2.26 ± 0.78 ^a,z^	3.39 ± 0.14 ^b,z^	2.79 ± 0.39 ^a,b,z^	3.42 ± 0.25 ^b,y^
8	8.86 ± 1.63 ^b,c,y^	10.58 ± 0.34 ^d,x^	9.55 ± 0.30 ^c,d,y^	7.21 ± 1.15 ^a,y^	8.02 ± 0.95 ^a,b,x^
12	9.04 ± 2.57 ^a,b,y^	9.32 ± 1.33 ^a,b,y^	11.53 ± 1.46 ^b,x^	7.01 ± 1.30 ^a,y^	8.27 ± 1.73 ^a,x^

C: sodium ascorbate control; Lva: 2.5% “verdejo” lees; Lvb: 5% “verdejo” lees; Lpa: 2.5% “palomino” lees; Lpb: 5% “palomino” lees. Means within a row with different superscript letters (^a–d^) are significantly different (*p* < 0.05) as a function of sample. Means within a column with different superscript letters (^z–w^) are significantly different (*p* < 0.05) as a function of storage.

**Table 4:** Concentration (mean ± standard deviation) of the most relevant volatile compounds (ng/g_DM_) in deer burgers at 0 and 12 days of chilled storage.

Compound	Day 0					Day 12				
	C	Lva	Lvb	Lpa	Lpb	C	Lva	Lvb	Lpa	Lpb
Hexanal	146.9 ± 54.3 ^a^	227.4 ± 57.4 ^a,b^	323.6 ± 58.7 ^b^	198.6 ± 28.2 ^a^	218.2 ± 33.6 ^a,b^	247.3 ± 9.0 ^b^	162.3 ± 12.8 ^a^	248.4 ± 30.2 ^b^	229.6 ± 43.7 ^b^	279.0 ± 44.3 ^b^
Octanal	26.9 ± 9.0 ^b^	11.2 ± 4.3 ^a,z^	21.5 ± 4.3 ^a,b^	14.5 ± 2.1 ^a,b^	15.2 ± 2.6 ^a,b,z^	44.2 ± 3.0 ^c^	21.3 ± 4.6 ^a,b,y^	25.6 ± 0.8 ^b^	15.6 ± 6.3 ^a^	27.5 ± 0.5 ^b,y^
2-Heptenal	36.2 ± 11.3 ^a^	50.3 ± 1.6 ^a^	118.3 ± 27.6 ^b^	42.9 ± 8.2 ^a^	61.6 ± 13.8 ^a^	42.3 ± 2.3 ^a^	47.2 ± 1.5 ^a^	65.8 ± 1.1 ^c^	55.8 ± 7.2 ^b^	66.3 ± 2.8 ^c^
Nonanal	51.2 ± 8.8 ^b,c,z^	37.5 ± 2.3 ^a,b,z^	59.5 ± 7.2 ^c^	34.0 ± 7.9 ^a,z^	38.1 ± 2.0 ^a,b,z^	87.2 ± 8.6 ^b,y^	53.9 ± 6.4 ^a,y^	74.3 ± 0.5 ^a,b^	73.0 ± 12.8 ^a,b,y^	74.0 ± 11.7 ^a,b,y^
(E)-2-Octenal	26.2 ± 2.4 ^a,z^	21.9 ± 7.0 ^a,z^	63.1 ± 13.3 ^b^	26.9 ± 5.2 ^a,y^	34.7 ± 4.8 ^a,z^	50.4 ± 3.0 ^y^	41.2 ± 3.2 ^y^	62.9 ± 13.9	59.7 ± 13.8 ^z^	64.0 ± 4.9 ^y^
(E)-2-Nonenal	23.3 ± 7.2 ^a^	15.9 ± 3.1 ^a^	49.0 ± 8.5 ^b^	18.6 ± 3.7 ^a^	28.5 ± 4.8 ^a^	25.5 ± 0.6 ^a,b^	17.7 ± 2.0 ^a^	32.0 ± 3.9 ^b,c^	20.7 ± 9.6 ^a,b^	38.9 ± 5.6 ^c^
2,4-Nonadienal	13.1 ± 4.9 ^a^	12.4 ± 3.0 ^a^	39.2 ± 7.3 ^b^	13.2 ± 3.3 ^a^	23.0 ± 4.2 ^a^	16.7 ± 0.6 ^a,b^	14.2 ± 2.1 ^a^	24.5 ± 6.2 ^b^	12.3 ± 3.0 ^a^	32.6 ± 6.7 ^c^
(E, E)-2,4-Decadienal	5.4 ± 2.9 ^a^	5.2 ± 1.3 ^a^	16.6 ± 2.1 ^b,y^	5.9 ± 2.6 ^a^	8.6 ± 1.8 ^a,z^	6.4 ± 2.3 ^a^	5.8 ± 0.8 ^a^	9.2 ± 2.5 ^a,z^	7.6 ± 1.5 ^a^	12.7 ± 1.8 ^b,y^
(E, Z)-2,4-Decadienal	8.2 ± 1.0 ^a,z^	13.4 ± 2.5 ^a,b^	40.9 ± 8.3 ^c^	13.9 ± 3.8 ^a,b^	22.7 ± 5.0 ^b,z^	20.1 ± 5.2 ^a,y^	18.1 ± 4.3 ^a^	29.3 ± 8.7 ^a^	21.8 ± 4.0 ^a^	47.3 ± 0.8 ^b,y^
1-Octen-3-one	3.2 ± 1.1 ^a^	4.0 ± 1.5 ^a^	10.6 ± 1.8 ^b^	3.6 ± 0.8 ^a,z^	5.3 ± 1.4 ^a^	5.8 ± 1.6	5.1 ± 0.4	7.6 ± 0.3	6.3 ± 1.0 ^y^	7.7 ± 1.5
1-Pentanol	2.4 ± 0.6 ^a,z^	3.3 ± 1.4 ^a,b,z^	5.4 ± 1.4 ^b,z^	3.4 ± 0.3 ^a,b,z^	3.1 ± 0.4 ^a,b,z^	13.6 ± 1.7 ^b,y^	7.0 ± 1.5 ^a,y^	9.5 ± 0.3 ^a,y^	9.1 ± 1.9 ^a,y^	10.2 ± 2.3 ^a,y^
4-Heptanol	1.8 ± 0.3 ^z^	8.0 ± 3.9	5.6 ± 1.0 ^y^	6.5 ± 4.0	2.6 ± 0.5	5.2 ± 0.5 ^b,y^	3.3 ± 0.4 ^a^	2.9 ± 0.5 ^a,z^	2.3 ± 1.0 ^a^	2.4 ± 0.3 ^a^
1-Hexanol	1.1 ± 0.4 ^a,z^	2.7 ± 2.0 ^a,b,z^	4.9 ± 0.6 ^b,z^	2.9 ± 0.3 ^a,b,z^	4.7 ± 1.3 ^b,z^	131.5 ± 21.7 ^y^	94.0 ± 12.2 ^y^	105.8 ± 16.6 ^y^	111.7 ± 26.1 ^y^	129.6 ± 21.4 ^y^
1-Octen-3-ol	24.3 ± 3.8 ^a,z^	33.4 ± 2.5 ^a,z^	53.6 ± 12.4 ^b^	29.0 ± 5.3 ^a,z^	28.4 ± 5.2 ^a,z^	60.8 ± 6.9 ^y^	62.4 ± 6.8 ^y^	70.8 ± 1.0	74.8 ± 12.8 ^y^	74.2 ± 16.2 ^y^
2-Ethyl-1-hexanol	4.7 ± 0.3 ^a,z^	29.3 ± 7.4 ^b^	32.5 ± 17.0 ^b^	15.9 ± 4.7 ^a,b^	12.8 ± 2.5 ^a,b^	52.2 ± 4.7 ^c,y^	24.9 ± 0.5 ^b^	22.2 ± 5.3 ^a,b^	20.9 ± 0.7 ^a,b^	14.6 ± 3.0 ^a^
1-Octanol	11.6 ± 3.9 ^a,z^	9.7 ± 2.7 ^a,z^	17.3 ± 3.2 ^b,z^	8.8 ± 1.4 ^a,z^	10.0 ± 1.4 ^a,z^	36.6 ± 3.5 ^c,y^	23.5 ± 3.2 ^a,b,y^	28.1 ± 0.1 ^b,y^	17.9 ± 4.0 ^a,y^	23.4 ± 3.1 ^a,b,y^
(E)-2-Octen-1-ol	nd ^z^	nd ^z^	nd ^z^	nd ^z^	nd ^z^	23.8 ± 3.9 ^y^	18.2 ± 2.0 ^y^	21.2 ± 5.8 ^y^	18.4 ± 5.4 ^y^	22.4 ± 5.1 ^y^
1-Nonanol	nd ^z^	nd ^z^	nd ^z^	nd ^z^	nd ^z^	8.7 ± 1.0 ^y^	8.3 ± 2.2 ^y^	9.8 ± 2.3 ^y^	7.3 ± 2.1 ^y^	11.2 ± 0.5 ^y^
Pentanoic acid	0.2 ± 0.1 ^a^	0.5 ± 0.2 ^a,z^	1.4 ± 0.1 ^c,z^	0.5 ± 0.1 ^a,z^	1.0 ± 0.2 ^b,z^	1.7 ± 1.1	2.4 ± 1.0 ^y^	2.8 ± 0.2 ^y^	3.5 ± 1.2 ^y^	2.5 ± 0.3 ^y^
Hexanoic acid	6.8 ± 2.6 ^a,z^	18.4 ± 4.4 ^a,z^	64.0 ± 10.5 ^b,z^	26.1 ± 5.2 ^a,z^	56.0 ± 24.9 ^b^	59.8 ± 12.4 ^a,y^	81.5 ± 6.2 ^a,y^	100.0 ± 1.12 ^a,b,y^	125.5±34.5 ^a,b,y^	76.6 ± 8.5 ^a^
Octanoic acid	8.0 ± 1.6 ^a,z^	197.9 ± 54.2 ^a^	952.1 ± 96.1 ^b,y^	223.1 ± 39.9 ^a,z^	824.4 ± 267.3 ^b^	16.1 ± 1.5 ^a,y^	250.6 ± 18.0 ^b^	581.9 ± 73.8 ^c,z^	323.0 ± 37.9 ^b,y^	762.3 ± 34.7 ^d^
Decanoic acid	7.7 ± 1.3 ^a,z^	316.5 ± 50.4 ^b,z^	1510.9 ± 236.1 ^d,y^	327.3 ± 41.2 ^b,z^	848.4 ± 101.2 ^c,z^	18.2 ± 3.8 ^a,y^	515.5 ± 82.7 ^b,y^	979.6 ± 158.7 ^c,z^	557.9 ± 42.0 ^b,y^	1270.2 ± 188.5 ^d,y^
Ethyl hexanoate	6.5 ± 2.5 ^a,z^	34.9 ± 7.7 ^b,z^	55.8 ± 9.2 ^c^	28.4 ± 6.1 ^b,z^	39.8 ± 4.2 ^b,z^	97.3 ± 18.8 ^y^	109.8 ± 9.7 ^y^	124.6 ± 32.1	142.0 ± 34.0 ^y^	93.7 ± 5.5 ^y^
Ethyl octanoate	7.6 ± 2.3 ^a,z^	276.1 ± 38.6 ^a^	1290.9 ± 34.0 ^b,y^	328.0 ± 57.7 ^a^	1289.0 ± 392.5 ^b^	19.8 ± 2.6 ^a,y^	249.4 ± 52.9 ^b^	430.6 ± 78.9 ^c,z^	324.0 ± 3.0 ^b,c^	626.0 ± 122.5 ^d^
Methyl decanoate	nd ^a^	3.9 ± 1.2 ^a^	14.7 ± 0.9 ^b,z^	3.8 ± 0.7 ^a^	12.5 ± 4.2 ^b^	nd ^a^	2.7 ± 0.9 ^b^	5.4 ± 1.3 ^c,y^	2.1 ± 0.8 ^b^	8.1 ± 1.2 ^d^
Hexyl hexanoate	nd ^z^	nd ^z^	nd ^z^	nd ^z^	nd ^z^	4.7 ± 1.5 ^y^	2.9 ± 0.9 ^y^	4.2 ± 1.8 ^y^	4.2 ± 2.0 ^y^	4.2 ± 1.1 ^y^
Ethyl decanoate	4.6 ± 2.6 ^a,z^	3161.4 ± 576.0 ^a,y^	13,321.0 ± 1281.4 ^b,y^	3201.1 ± 478.0 ^a,y^	11,788.6 ± 4044.9 ^b^	42.5 ± 2.4 ^a,y^	1190.7 ± 266.9 ^b,z^	2884.3 ± 1011.4 ^c,z^	1546.1 ± 271.9 ^b,z^	5680.9 ± 430.1 ^d^
3-Methylbutyl octanoate	nd ^a^	35.3 ± 6.3 ^b,y^	160.4 ± 23.0 ^d,z^	36.7 ± 4.4 ^b^	102.6 ± 15.9 ^c^	nd ^a^	17.2 ± 4.4 ^a,b,z^	91.6 ± 22.5 ^c,y^	31.5 ± 3.1 ^b^	104.4 ± 4.1 ^c^
Propyl decanoate	nd ^a^	2.9 ± 0.2 ^a^	12.6 ± 2.3 ^b,y^	2.8 ± 0.1 ^a^	9.9 ± 3.3 ^b^	nd ^a^	2.6 ± 1.1 ^b^	5.5 ± 1.2 ^c,z^	2.9 ± 0.6 ^b^	9.2 ± 0.7 ^d^
Butyl decanoate	nd ^a^	2.7 ± 0.5 ^a^	12.2 ± 2.4 ^b,y^	2.7 ± 0.3 ^a^	9.1 ± 3.5 ^b^	nd ^a^	2.3 ± 0.9 ^b^	4.9 ± 1.4 ^c,z^	2.6 ± 0.7 ^b^	9.6 ± 0.6 ^d^
Methyl dodecanoate	nd ^a^	0.3 ± 0.0 ^a,z^	2.9 ± 0.4 ^b,y^	0.4 ± 0.1 ^a,z^	2.7 ± 0.8 ^b^	nd ^a^	1.2 ± 0.1 ^b,y^	1.4 ± 0.5 ^b,z^	1.0 ± 0.3 ^b,y^	2.6 ± 0.4 ^c^
3-Methylbutyl decanoate	nd ^a^	40.1 ± 7.6 ^b^	182.2 ± 35.2 ^d,y^	37.5 ± 5.4 ^b^	112.0 ± 15.1 ^c^	nd ^a^	36.3 ± 11.5 ^b^	81.1 ± 18.8 ^c,z^	44.8 ± 3.5 ^b^	137.2 ± 35.7 ^d^
p-Cymene	0.3 ± 0.2 ^y^	2.2 ± 1.9	1.4 ± 0.2 ^y^	0.8 ± 0.1 ^y^	1.2 ± 0.4	0.8 ± 0.1 ^a,z^	0.8 ± 0.0 ^a^	1.2 ± 0.1 ^c,z^	0.9 ± 0.1 ^a,z^	1.1 ± 0.0 ^b^
Trimethyl benzene	0.4 ± 0.1 ^a,y^	0.9 ± 0.3 ^b,c,y^	1.2 ± 0.1 ^c,y^	0.7 ± 0.1 ^a,b,y^	1.0 ± 0.1 ^b,c^	0.4 ± 0.1 ^a,z^	0.7 ± 0.0 ^b,z^	0.9 ± 0.1 ^c,z^	0.7 ± 0.1 ^b,z^	1.1 ± 0.1 ^d^
Benzaldehyde	18.3 ± 5.6 ^a,z^	17.4 ± 4.7 ^a,z^	40.5 ± 6.7 ^b,z^	17.5 ± 3.3 ^a,z^	27.5 ± 6.6 ^a,z^	64.1 ± 1.5 ^a,y^	93.9 ± 19.5 ^a,b,y^	75.5 ± 8.1 ^a,y^	114.9 ± 15.9 ^b,y^	93.7 ± 6.0 ^a,b,y^
Phenylethyl alcohol	1.2 ± 0.7 ^a,z^	29.0 ± 2.1 ^b^	145.3 ± 18.1 ^d,y^	39.4 ± 6.7 ^b^	105.9 ± 12.0 ^c^	30.2 ± 3.7 ^a,y^	41.0 ± 6.1 ^a^	92.0 ± 2.2 ^b,z^	51.3 ± 7.1 ^a^	119.3 ± 20.2 ^c^
Carbon disulfide	0.6 ± 0.1 ^a^	1.3 ± 0.4 ^b^	1.0 ± 0.2 ^a,b^	0.5 ± 0.1 ^a^	1.0 ± 0.3 ^a,b^	1.4 ± 0.5 ^b^	0.7 ± 0.3 ^a,b^	0.7 ± 0.2 ^a,b^	0.5 ± 0.2 ^a^	1.0 ± 0.4 ^a,b^
5-Methyl thiazole	nd ^z^	nd ^z^	nd ^z^	nd ^z^	nd ^z^	11.3 ± 4.0 ^a,y^	9.7 ± 0.8 ^a,z^	38.4 ± 16.7 ^b,y^	8.9 ± 2.7 ^a,y^	20.0 ± 9.7 ^a,y^
2-Pentyl furan	12.1 ± 3.1 ^a,z^	19.7 ± 4.5 ^a,z^	29.5 ± 6.4 ^b^	12.9 ± 1.7 ^a,z^	14.8 ± 1.2 ^a,z^	23.6 ± 3.9 ^y^	26.7 ± 3.0 ^y^	38.4 ± 6.7	37.6 ± 4.9 ^y^	36.7 ± 8.0 ^y^
Total concentrations of the main groups of compounds *										
∑ Aldehydes	422.7 ± 91.3 ^a,z^	450.0 ± 53.7 ^a^	853.0 ± 155.9 ^b^	416.2 ± 65.4 ^a^	520.5 ± 75.9 ^a,z^	685.9 ± 31.5 ^b,c,y^	488.3 ± 32.6 ^a^	660.0 ± 75.9 ^b,c^	570.4 ± 100.6 ^a,b^	758.4 ± 74.4 ^c,y^
∑ Ketones	30.9 ± 7.3	27.5 ± 7.0 ^z^	37.1 ± 3.0 ^z^	23.4 ± 3.2 ^z^	27.1 ± 5.9 ^z^	38.2 ± 6.3 ^a^	43.0 ± 1.6 ^a,y^	58.3 ± 6.3 ^b,y^	55.7 ± 6.3 ^b,y^	47.1 ± 5.6 ^a,b,y^
∑ Alcohols	66.5 ± 13.1 ^a,z^	107.1 ± 13.8 ^a,z^	142.4 ± 35.1 ^b,z^	84.3 ± 12.3 ^a,z^	79.2 ± 8.9 ^a,z^	379.8 ± 48.4 ^b,y^	267.6 ± 28.8 ^a,y^	305.9 ± 18.8 ^a,b,y^	301.0 ± 42.9 ^a,b,y^	323.7 ± 42.6 ^a,b,y^
∑ Hydrocarbons	26.0 ± 9.8 ^a^	26.4 ± 8.5 ^a,z^	48.5 ± 11.7 ^b^	25.5 ± 4.8 ^a,z^	25.5 ± 2.2 ^a,z^	36.1 ± 3.8 ^a^	40.6 ± 1.5 ^a,b,y^	52.6 ± 8.1 ^b^	54.8 ± 10.5 ^b,y^	45.2 ± 4.3 ^a,b,y^
∑ Acids	37.5 ± 9.0 ^a,z^	571.0 ± 102.1 ^b,z^	2765.7 ± 384.0 ^d,y^	627.7 ± 92.4 ^b,z^	1844.0 ± 340.9 ^c^	138.8 ± 14.3 ^a,y^	925.8 ± 123.0 ^b,y^	1828.2 ± 250.1 ^c,z^	1092.1 ± 124.4 ^b,y^	2278.9 ± 227.5 ^d^
∑ Esters	18.9 ± 6.5 ^a,z^	4289.2 ± 753.4 ^a,y^	18,192.2 ± 1860.4 ^b,y^	4468.6 ± 664.5 ^a,y^	16,072.1 ± 5197.0 ^b^	196.9 ± 27.6 ^a,y^	2140.8 ± 276.4 ^b,z^	4885.7 ± 1280.0 ^c,z^	3020.2 ± 401.0 ^b,z^	8856.2 ± 547.3 ^d^
∑ Benzenic compounds	25.5 ± 7.6 ^a,z^	57.3 ± 10.0 ^b,z^	210.5 ± 28.3 ^d^	65.3 ± 11.4 ^b,z^	141.7 ± 15.4 ^c,z^	126.0 ± 7.1 ^a,y^	158.9 ± 27.6 ^a,b,y^	203.3 ± 11.5 ^b^	192.2 ± 25.4 ^b,y^	251.7 ± 21.1 ^c,y^
∑ Sulfur compounds	0.6 ± 0.1 ^a,z^	1.3 ± 0.4 ^b,z^	1.0 ± 0.2 ^a,b,z^	0.5 ± 0.1 ^a,z^	1.0 ± 0.3 ^a,b^	12.7 ± 3.8 ^a,y^	10.3 ± 1.1 ^a,y^	39.1 ± 16.6 ^b,y^	9.4 ± 2.9 ^a,y^	21.1 ± 9.6 ^a^
∑ Furanic compounds	14.1 ± 3.0 ^a,z^	23.1 ± 6.2 ^a^	34.7 ± 7.9 ^b^	15.2 ± 2.5 ^a,z^	19.7 ± 0.9 ^a^	26.2 ± 4.1 ^a,y^	30.3 ± 3.1 ^a,b^	43.6 ± 7.0 ^b^	40.6 ± 5.1 ^a,b,y^	40.5 ± 9.0 ^a,b^

* Sum of the concentrations of the volatile compounds shown in Table S1 (supplementary material) grouped into chemical families. C: sodium ascorbate control; Lva: 2.5% “verdejo” lees; Lvb: 5% “verdejo” lees; Lpa: 2.5% “palomino” lees; Lpb: 5% “palomino” lees; DM: dry matter. Means within a row with different superscript letters (^a–d^) are significantly different (*p* < 0.05) as a function of sample within the same day. Means within a row with different superscript letters (^z–y^) are significantly different (*p* < 0.05) as a function of storage within the same sample.

**Table 5:** Result of principal component analysis applied to volatile data obtained from deer burgers at 0 and 12 days of chilled storage. Volatile compounds more correlated with the two first Principal Component and their loading.

Principal Component	Compounds	Loadings
PC 1	Butyl decanoate	0.981
	Propyl decanoate	0.967
	3-Methylbutyl decanoate	0.967
	Octanoic acid	0.964
	Phenylethyl alcohol	0.956
	3-Methylbutyl octanoate	0.955
	Methyl dodecanoate	0.949
	Methyl decanoate	0.945
PC 2	Benzaldehyde	0.956
	1-Hexanol	0.942
	1-Nonanol	0.919
	Ethyl-hexanoate	0.915
	Pentanoic acid	0.909
	(E)-2-octen-1-ol	0.908
	1-Octen-3-ol	0.906
	Hexyl hexanoate	0.904
	Hexanoic acid	0.899

## Supplementary Materials

The following are available online at https://www.mdpi.com/2076-3921/9/8/687/s1, Table S1. Concentration (mean ± standard deviation) of volatile compounds (ng/g_DM_) in deer burgers at 0 and 12 days of chilled storage.

## References

[1] Soriano A., Murillo P., Perales M., Sánchez-García C., Murillo J.A., García Ruíz A. (2020). Nutritional quality of wild Iberian red deer (*Cervus elaphus hispanicus*) meat: Effects of sex and hunting period. Meat Sci..

[2] DeMartini E., Vecchiato D., Tempesta T., Gaviglio A., Viganò R., Eugenio D., Vecchiato D., Tiziano T., Anna A.G., Roberto V. (2018). Consumer preferences for red deer meat: A discrete choice analysis considering attitudes towards wild game meat and hunting. Meat Sci..

[3] Lund M.N., Hviid M.S., Skibsted L.H. (2007). The combined effect of antioxidants and modified atmosphere packaging on protein and lipid oxidation in beef patties during chill storage. Meat Sci..

[4] Karre L., Lopez K., Getty K.J.K. (2013). Natural antioxidants in meat and poultry products. Meat Sci..

[5] Devesa-Rey R., Vecino X., Varela-Alende J.L., Barral M.T., Cruz J.M., Moldes A.B. (2011). Valorization of winery waste vs. the costs of not recycling. Waste Manag..

[6] Ruggieri L., Cadena E., Martínez-Blanco J., Gasol C.M., Rieradevall J., Gabarrell X., Gea T., Sort X., Sánchez A. (2009). Recovery of organic wastes in the Spanish wine industry. Technical, economic and environmental analyses of the composting process. J. Clean. Prod..

[7] Teixeira A., Baenas N., Domínguez-Perles R., Barros A.R., Rosa E.A., Moreno D.A., Garcia-Viguera C. (2014). Natural Bioactive Compounds from Winery By-Products as Health Promoters: A Review. Int. J. Mol. Sci..

[8] Dimou C., Kopsahelis N., Papadaki A., Papanikolaou S., Kookos I.K., Mandala I.G., Koutinas A. (2015). Wine lees valorization: Biorefinery development including production of a generic fermentation feedstock employed for poly(3-hydroxybutyrate) synthesis. Food Res. Int..

[9] Jurčević I.L., Dora M., Guberović I., Petras M., Brnčić S.R., Đikić D., Landeka I., Rimac S. (2017). Polyphenols from Wine Lees as a Novel Functional Bioactive Compound in the Protection against Oxidative Stress and Hyperlipidaemia. Food Technol. Biotechnol..

[10] Rodríguez-Bencomo J.J., Andújar-Ortiz I., Moreno-Arribas M.V., Simo C., González J., Chana A., Dávalos J.Z., Ángeles Pozo-Bayón M. (2014). Impact of Glutathione-Enriched Inactive Dry Yeast Preparations on the Stability of Terpenes during Model Wine Aging. J. Agric. Food Chem..

[11] Hwang J.-Y., Shyu Y.-S., Hsu C.-K. (2009). Grape wine lees improves the rheological and adds antioxidant properties to ice cream. LWT Food Sci. Technol..

[12] Sharma A.K., Kumar R., Azad Z.R.A.A., Adsule P.G. (2015). Use of fine wine lees for value addition in ice cream. J. Food Sci. Technol..

[13] Garrido M.D., Auqui M., Martí N., Linares M.B. (2011). Effect of two different red grape pomace extracts obtained under different extraction systems on meat quality of pork burgers. LWT Food Sci. Technol..

[14] Selani M.M., Contreras-Castillo C.J., Shirahigue L.D., Gallo C.R., Plata-Oviedo M.S., Montes-Villanueva N.D. (2011). Wine industry residues extracts as natural antioxidants in raw and cooked chicken meat during frozen storage. Meat Sci..

[15] García-Lomillo J., González-San José M.L., Del Pino-García R., Rivero-Pérez M.D., Muñiz-Rodríguez P. (2014). Antioxidant and Antimicrobial Properties of Wine Byproducts and Their Potential Uses in the Food Industry. J. Agric. Food Chem..

[16] Soriano A., Alañón M.E., Alarcón M., García-Ruíz A., Díaz-Maroto M.C., Pérez-Coello M.S. (2018). Oak wood extracts as natural antioxidants to increase shelf life of raw pork patties in modified atmosphere packaging. Food Res. Int..

[17] Serrano A., Cofrades S., Jiménez-Colmenero F. (2006). Characteristics of restructured beef steak with different proportions of walnut during frozen storage. Meat Sci..

[18] Ganhão R., Morcuende D., Estévez M. (2010). Protein oxidation in emulsified cooked burger patties with added fruit extracts: Influence on colour and texture deterioration during chill storage. Meat Sci..

[19] Hierro E., De La Hoz L., Ordóñez J.A. (2004). Headspace volatile compounds from salted and occasionally smoked dried meats (cecinas) as affected by animal species. Food Chem..

[20] Mazza G., Fukumoto L., Delaquis P., Girard B., Ewert B. (1999). Anthocyanins, phenolics, and color of Cabernet Franc, Merlot, and Pinot Noir wines from British Columbia. J. Agric. Food Chem..

[21] Vivas N., Glories Y., Lagune L., Saucier C., Augustin M. (1994). Estimation du degré de polymérisation des procyanidines du raisin et du vin par la méthode au p-dimethylaminocinnamaldéhyde. OENO One.

[22] Yin M.C., Faustman C. (1993). Influence of temperature, pH, and phospholipid composition upon the stability of myoglobin and phospholipid: A liposome model. J. Agric. Food Chem..

[23] Lorenzo J.M., Sineiro J., Amado I.R., Franco D. (2014). Influence of natural extracts on the shelf life of modified atmosphere-packaged pork patties. Meat Sci..

[24] Andrés A.I., Petrón M.J., Adámez J.D., López M., Timón M.L. (2017). Food by-products as potential antioxidant and antimicrobial additives in chill stored raw lamb patties. Meat Sci..

[25] Vergara H., Gallego L., García A.J., Landete-Castillejos T. (2003). Conservation of *Cervus elaphus* meat in modified atmospheres. Meat Sci..

[26] Soriano A., Montoro V., Vicente J., Sánchez-Migallón B.F., Benítez S., Utrilla M.C., García Ruiz A. (2016). Influence of evisceration time and carcass ageing conditions on wild venison quality. Preliminary study. Meat Sci..

[27] Kim H.S., Chin K.B. (2017). Evaluation of Antioxidative Activity of Various Levels of Ethanol Extracted Tomato Powder and Application to Pork Patties. Korean J. Food Sci. Anim. Resour..

[28] Chouliara E., Karatapanis A., Savvaidis I.N., Kontominas M.G. (2007). Combined effect of oregano essential oil and modified atmosphere packaging on shelf-life extension of fresh chicken breast meat, stored at 4 °C. Food Microbiol..

[29] Mancini S., Preziuso G., Dal Bosco A., Roscini V., Szendrő Z., Fratini F., Paci G. (2015). Effect of turmeric powder (Curcuma longa L.) and ascorbic acid on physical characteristics and oxidative status of fresh and stored rabbit burgers. Meat Sci..

[30] Zamuz S., López-Pedrouso M., Barba F.J., Lorenzo J.M., Domínguez H., Franco D. (2018). Application of hull, bur and leaf chestnut extracts on the shelf-life of beef patties stored under MAP: Evaluation of their impact on physicochemical properties, lipid oxidation, antioxidant, and antimicrobial potential. Food Res. Int..

[31] Primo E. (1998). Oleaginosas. Grasas Animales. Grasas Plásticas. Química de los Alimentos.

[32] Zhang W., Xiao S., Ahn D.U. (2013). Protein Oxidation: Basic Principles and Implications for Meat Quality. Crit. Rev. Food Sci. Nutr..

[33] García-Lomillo J., González-SanJosé M.L., Skibsted L.H., Jongberg S. (2016). Effect of skin wine pomace and sulfite on protein oxidation in beef patties during high oygen atmosphere storage. Food Bioprocess Technol..

[34] Ergezer H., Serdaroğlu M. (2018). Antioxidant potential of artichoke (Cynara scolymus L.) byproducts extracts in raw beef patties during refrigerated storage. J. Food Meas. Charact..

[35] Mottram D.S. (1998). Flavour formation in meat and meat products: A review. Food Chem..

[36] Olivares A., Dryahina K., Španěl P., Flores M. (2012). Rapid detection of lipid oxidation in beef muscle packed under modified atmosphere by measuring volatile organic compounds using SIFT-MS. Food Chem..

[37] Okabe Y., Watanabe A., Shingu H., Kushibiki S., Hodate K., Ishida M., Ikeda S., Takeda T. (2002). Effects of α-tocopherol level in raw venison on lipid oxidation and volatiles during storage. Meat Sci..

[38] Shahidi F., Shahidi F. (1994). Flavor of meat and meat products, an overview. Flavor of Meat and Meat Products.

[39] García-Lomillo J., González-San José M.L., Del Pino-García R., Ortega-Heras M., Muñiz-Rodríguez P. (2017). Antioxidant effect of seasonings derived from wine pomace on lipid oxidation in refrigerated and frozen beef patties. LWT Food Sci. Technol..

[40] DeMan J.H. (1990). Lipids in Principles of Food Chemistry.

[41] Marco A., Navarro J.L., Flores M. (2007). Quantitation of Selected Odor-Active Constituents in Dry Fermented Sausages Prepared with Different Curing Salts. J. Agric. Food Chem..

[42] Paleari M.A., Moretti V.M., Beretta G., Mentasti T., Bersani C. (2003). Cured products from different animal species. Meat Sci..

[43] Price A., Díaz P., Bañón S., Garrido M.D. (2013). Natural extracts versus sodium ascorbate to extend the shelf life of meat-based ready-to-eat meals. Food Sci. Technol. Int..

[44] Ortuño J., Serrano R., Bañón S. (2016). Use of dietary rosemary diterpenes to inhibit rancid volatiles in lamb meat packed under protective atmosphere. Animal.

[45] Andrés A.I., Cava R., Ventanas S., Muriel E., Ruiz J., Ruiz-Carrascal J. (2007). Effect of salt content and processing conditions on volatile compounds formation throughout the ripening of Iberian ham. Eur. Food Res. Technol..

[46] Sabio E., Vidal-Aragón M.C., Bernalte M.J., Gata J.L. (1998). Volatile compounds present in six types of dry-cured ham from south European countries. Food Chem..

[47] Ramírez R., Cava R. (2007). Volatile Profiles of Dry-Cured Meat Products from Three Different Iberian X Duroc Genotypes. J. Agric. Food Chem..

[48] Martín A., Córdoba J.J., Aranda E., Córdoba M.G., Asensio M.A. (2006). Contribution of a selected fungal population to the volatile compounds on dry-cured ham. Int. J. Food Microbiol..

[49] Rodríguez-Carpena J.-G., Morcuende D., Estévez M. (2011). Avocado by-products as inhibitors of color deterioration and lipid and protein oxidation in raw porcine patties subjected to chilled storage. Meat Sci..

